# Supplementary material for: Splice-Junction-Based Mapping of Alternative Isoforms in the Human Proteome
Source: Cell Rep. Author manuscript; Available in PMC 2020 Jan 15. (PMC6961840; doi:10.1016/j.celrep.2019.11.026)
Supplement: 3 [file NIHMS1546469-supplement-3.zip › DF2/PXD0010154/Lung-365-Q68EM7-ESPPKPKDPVSAAVPAPGR.pdf]

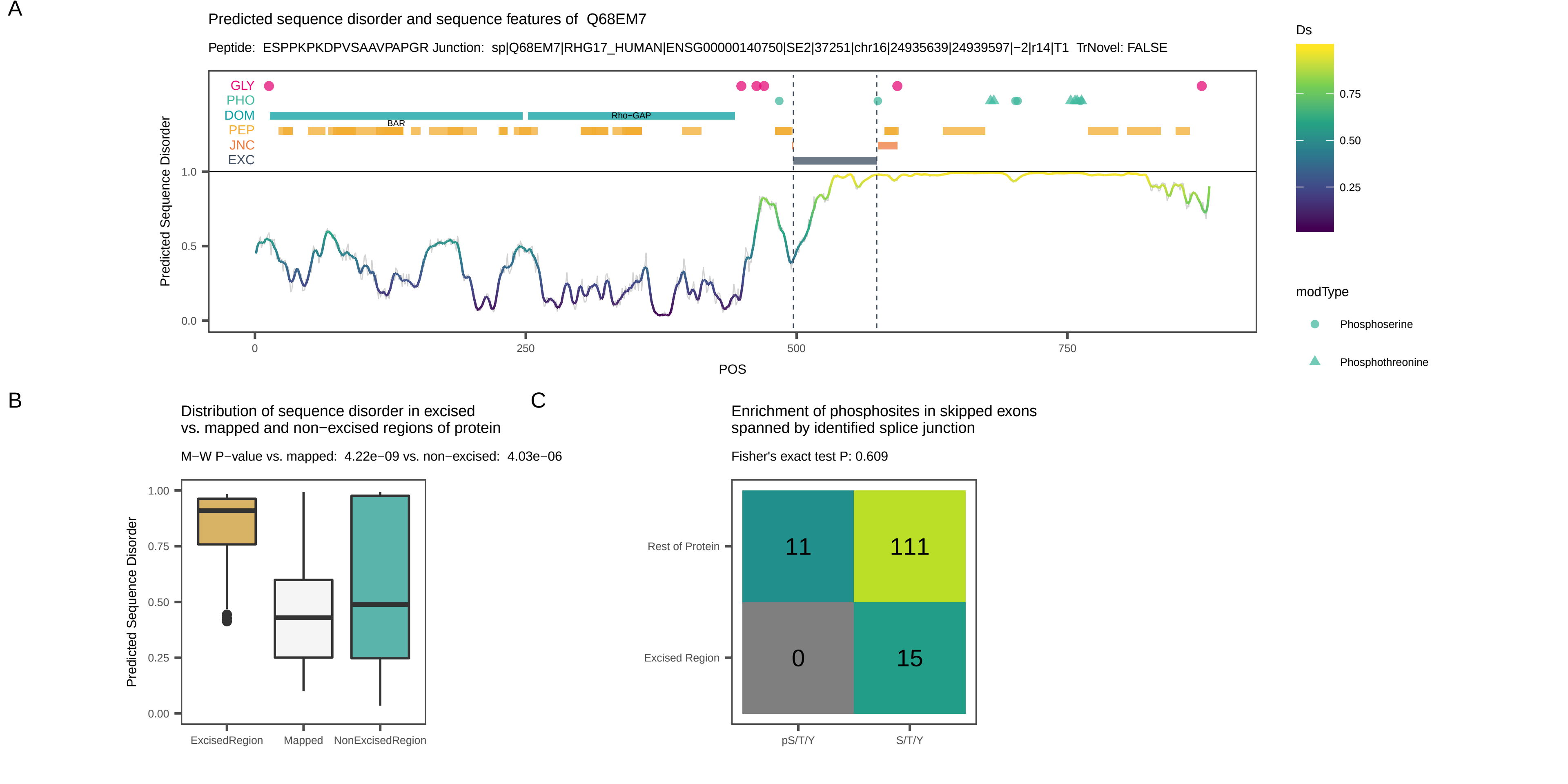
A
Predicted sequence disorder and sequence features of Q68EM7
Peptide: ESPPKPKDPVSAAVPAPGR Junction: sp|Q68EM7|RHG17_HUMAN|ENSG00000140750|SE2|37251|chr16|24935639|24939597|−2|r14|T1 TrNovel: FALSE
GLY
PHO
DOM
PEP
JNC
EXC
BAR
Rho−GAP
Predicted Sequence Disorder
1.0
0.5
0.0
0
250
500
750
POS
Ds
0.75
0.50
0.25
modType
Phosphoserine
Phosphothreonine
B
Distribution of sequence disorder in excised
vs. mapped and non−excised regions of protein
M−W P−value vs. mapped: 4.22e−09 vs. non−excised: 4.03e−06
Predicted Sequence Disorder
1.00
0.75
0.50
0.25
0.00
ExcisedRegion
Mapped
NonExcisedRegion
C
Enrichment of phosphosites in skipped exons
spanned by identified splice junction
Fisher's exact test P: 0.609
Rest of Protein
Excised Region
11
111
0
15
pS/T/Y
S/T/Y
